# AID–2×RBD27, an auxin-inducible degron-based Rab27 trapper that reversibly inhibits the function of Rab27A in melanocytes

**DOI:** 10.1242/jcs.263878

**Published:** 2025-06-10

**Authors:** Akira Sugawara, Yuto Maruta, Mitsunori Fukuda

**Affiliations:** Laboratory of Membrane Trafficking Mechanisms, Department of Integrative Life Sciences, Graduate School of Life Sciences, Tohoku University, Aobayama, Aoba-ku, Sendai, Miyagi 980-8578, Japan

**Keywords:** Auxin-inducible degron, 3D live-cell imaging, Melanosome transport, Rab27 effector domain, Reversible inhibition, Small GTPase Rab

## Abstract

Small GTPase Rabs are evolutionarily conserved regulators of intracellular membrane traffic and regulate multiple steps in membrane trafficking. Although various approaches have been used to identify the function(s) of individual Rabs, no simple tool that reversibly inhibits the function of Rab has ever been reported. Here, we developed a novel tool, named AID–2×RBD27 (auxin-inducible degron-tagged tandem Rab27-binding domain), that reversibly inhibits the function of Rab27 and then evaluated its usefulness by using Rab27A-mediated melanosome transport as a model. We showed that expression and degradation of AID–2×RBD27 in melanocytes caused reversible changes in melanosome distribution between perinuclear melanosome aggregation and peripheral melanosome dispersion. By performing 3D live-cell imaging in combination, we found that two types of anterograde melanosome transport are involved in peripheral melanosome dispersion, i.e. fast, long-range melanosome transport in the microtubule-enriched inner cellular region, especially in the dendrite, and slow, intermittent melanosome transport along the cortical actin filaments. Our new concept of an auxin-inducible degron–Rab-binding domain system would apply to all other Rabs as a means of investigating various Rab-mediated membrane trafficking events by reversibly inhibiting them.

## Teaser

**Highlighted Article:** In this study, we developed a novel auxin-inducible degron-based Rab27 trapper (named AID–2×RBD27) that can reversibly inhibit the function of Rab27A in melanosome transport in melanocytes.

## INTRODUCTION

Rab small GTPases are master regulators of intracellular membrane traffic that are conserved in all eukaryotes, and ∼60 different Rab isoforms are present in mammals (Stenmark, 2009; Hutagalung and Novick, 2011; Pfeffer, 2013; Barr, 2013; Homma et al., 2021). Rabs function as a molecular switch by cycling between a GDP-bound inactive form and a GTP-bound active form, which recruits its effector protein(s) to a specific organelle membrane and promotes various steps in membrane trafficking, including vesicle budding, transport, tethering, docking and fusion.

Various approaches – including small interference RNA (siRNA)-mediated knockdown, CRISPR/Cas9-mediated gene knockout, overexpression of a constitutively active (CA) or negative (CN) form of Rabs, and overexpression of a specific Rab-binding domain (RBD) – have often been used to analyze the function and physiological significance of Rabs at the cellular level (Fukuda, 2010). Although these approaches are powerful and useful for analyzing Rab functions, the tools used in the previous approaches have had several drawbacks. For example, because the tools are basically ‘irreversible’, additional experiments are needed to determine whether an observed phenotype(s) produced by the tools is directly related to inhibition or to promotion of the Rabs. siRNAs and guide RNAs sometimes have off-target effects, and thus appropriate rescue experiments to rule out the possibility of an off-target effect are necessary. Overexpression of an RBD as a dominant-negative construct, which can irreversibly trap endogenous active Rab and inhibit a Rab–effector interaction, necessitates additional control experiments, e.g. overexpression of an RBD mutant that lacks Rab binding ability, in order to demonstrate the specificity of the effect of the RBD on membrane traffic (e.g. RBD11 and mutant RBD11; Osaki et al., 2021). Moreover, overexpression of CA or CN Rab mutants would also irreversibly affect membrane traffic, even if the corresponding Rabs are not endogenously expressed. Thus, no easy-to-use tools that ‘reversibly’ inhibit Rab functions are currently available.

In this study, we developed a novel tool called AID–2×RBD27 (auxin-inducible degron-tagged tandem Rab27-binding domain) that reversibly inhibits the function of Rab27 and then validated its usefulness by using melanosome transport as a model (Marks and Seabra, 2001; Hume and Seabra, 2011). We used a Rab27 effector domain as an RBD specific for Rab27 (RBD27; Fukuda, 2013) in combination with auxin-inducible degron (AID) technology, in which AID-tagged proteins are rapidly degraded by the ubiquitin–proteasome pathway after treatment with auxin or its analog (Nishimura et al., 2009; Yesbolatova et al., 2020). The results showed that expression and degradation of AID–2×RBD27 in melanocytes reversibly inhibited actin-based melanosome transport, i.e. induction of perinuclear melanosome aggregation and recovery of peripheral melanosome distribution. We used this system to analyze anterograde melanosome transport by performing live-cell imaging of melanocytes that had or did not have a dendrite and evaluated the relative contribution of microtubule-dependent and actin-dependent movements in anterograde melanosome transport. Based on our results, we discuss the usefulness of the AID–RBD system for functional analyses of Rab small GTPases in membrane trafficking.

## RESULTS

### The synaptotagmin-like protein homology domain of Slp2-a is a specific Rab27-binding domain

**Fig. 1.**
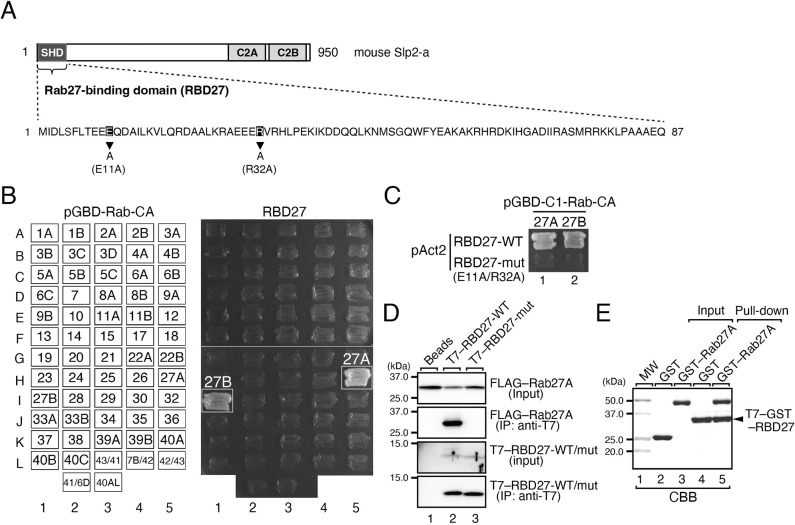
**RBD27 specifically recognized the Rab27 isoforms among the Rab family members.** (A) Schematic representation of mouse Slp2-a and the amino acid sequence of RBD27. The positions of the amino acid substitutions (E11A and R32A) in the Rab27A binding-defective mutant (RBD27-mut) are indicated by arrowheads. (B) Specific interactions between RBD27 and Rab27 isoforms (Rab27A and Rab27B), as revealed by yeast two-hybrid assays. Interactions were detected by growth of the yeast cells, and positive patches are boxed. (C) Loss of Rab27 binding ability of RBD27-mut (E11A/R32A) as revealed by yeast two-hybrid assays. (D) Interaction between RBD27-WT or -mut and Rab27A as revealed by co-immunoprecipitation assays using anti-T7 tag antibody-conjugated agarose beads. Image is representative of two experimental repeats. (E) Direct interaction between purified GST–Rab27A (or GST as a negative control) and anti-T7 tag-antibody-conjugated agarose beads coupled with T7–GST–RBD27 as revealed by pulldown assays. Proteins bound to the beads were detected by Coomassie Brilliant Blue (CBB) staining. Although several minor bands in addition to the major T7–GST–RBD27 band were often observed by CBB staining, all these bands were detected with anti-T7 tag antibody (data not shown), indicating that they are likely to be degradation products or post-translationally modified forms of T7–GST–RBD27. The positions of the molecular mass markers (kDa) are shown on the left in D and E. Data in D and E are representative of the data obtained in two and three independent experiments, respectively, and similar results were obtained in each experiment.

To develop a tool that reversibly inhibits the function of Rab small GTPases in intracellular membrane trafficking, we first focused on a specific Rab effector domain, the synaptotagmin-like protein (Slp) homology domain (SHD) of Slp2-a (also known as SYTL2), a known Rab27 effector protein, because previous studies have shown that the SHD of Slp2-a (referred to as RBD27; Fig. 1A) directly interacts with the active, GTP-bound form of Rab27 isoforms (i.e. Rab27A and Rab27B) with very high affinity [dissociation constant (*K*_D_)=∼13 nM] (Fukuda, 2006). We confirmed the specific interactions between RBD27 and Rab27 isoforms by yeast two-hybrid assays using 62 different CA Rab mutants as bait. As shown in Fig. 1B, wild-type RBD27 (RBD27-WT) interacted specifically with the Rab27 isoforms, but it did not recognize any of the other 60 Rabs. By contrast, a Rab27A-binding-defective mutant (E11A/R32A; named RBD27-mut; Kuroda and Fukuda, 2004) was incapable of binding to Rab27 (Fig. 1C). A specific interaction between RBD27 and Rab27 in mammalian cells was also confirmed by co-immunoprecipitation assays in COS-7 cells expressing T7-tagged RBD27-WT (or RBD27-mut) and FLAG-tagged Rab27A (Fig. 1D, second panel). Moreover, a direct interaction between RBD27 and Rab27 was demonstrated by *in vitro* direct binding assays using purified components (i.e. T7–GST–RBD27 and GST–Rab27A) (Fig. 1E, lane 5).

### Expression of 2×RBD27 in melanocytes causes perinuclear melanosome aggregation by trapping endogenous Rab27A on melanosomes

**Fig. 2.**
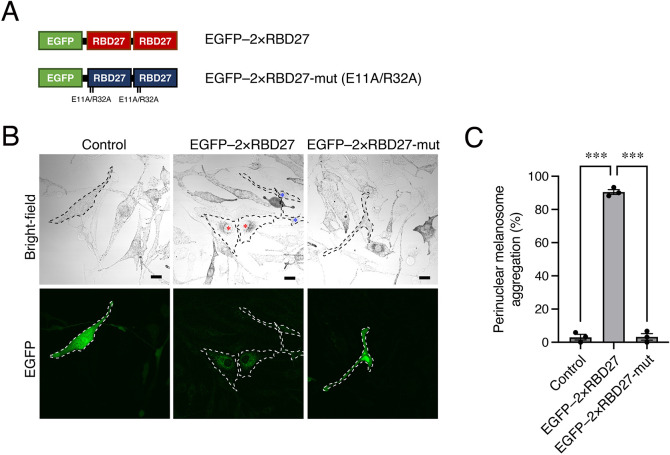
**Expression of 2×RBD27 in melan-a cells induced perinuclear melanosome aggregation.** (A) Schematic representation of EGFP-tagged 2×RBD27 and 2×RBD27-mut (E11A/R32A). (B) Typical images of melan-a cells (outlined with dashed lines) expressing EGFP alone (Control), EGFP–2×RBD27 or EGFP–2×RBD27-mut. The red asterisks indicate cells that exhibit perinuclear melanosome aggregation, whereas the blue asterisks indicate the cells that do not exhibit a phenotype, presumably owing to the low expression level of EGFP–2×RBD27 (i.e. insufficient dominant-negative effect of EGFP–2×RBD27). Scale bars: 20 μm. (C) The percentages of cells exhibiting perinuclear melanosome aggregation in B. Data represent the means±s.e.m. of data obtained in three independent experiments (*n*>25 cells in each experiment). ***P<0.001 (one-way ANOVA and Tukey's test).

Because 2×RBD11 (tandem RBD11) has previously been shown to have a stronger dominant-negative effect by trapping endogenous Rab11 in cultured cells than single RBD11 (Osaki et al., 2021), we prepared 2×RBD27-WT (unless otherwise specified, 2×RBD27 means 2×RBD27-WT) and 2×RBD27-mut (Fig. 2A) and evaluated their effects on actin-based melanosome transport, which is known to be specifically regulated by Rab27A, in melanocytes (Fukuda et al., 2002; Wu et al., 2002; Strom et al., 2002). As shown in Fig. 2B (red asterisks), expression of EGFP-tagged 2×RBD27 in melan-a cells (an immortal melanocyte cell line; Bennett et al., 1987) often caused perinuclear melanosome aggregation, a typical Rab27A-defective phenotype (Bahadoran et al., 2001; Hume et al., 2001). By contrast, neither EGFP alone nor EGFP–2×RBD27-mut affected peripheral melanosome distribution in melan-a cells. The results of a quantitative analysis showed that the percentage of cells exhibiting perinuclear melanosome aggregation was significantly higher in the EGFP–2×RBD27-expressing cells (∼90%) than in the EGFP-expressing cells or EGFP–2×RBD27-mut-expressing cells (∼5%; Fig. 2C). Moreover, EGFP fluorescence by 2×RBD27 was observed at the aggregated melanosomes in the perinucleus, whereas EGFP–2×RBD27-mut appeared to localize in the cytosol, strongly suggesting that 2×RBD27 specifically traps Rab27A on melanosomes (Fig. 2B). Taken together, these results indicated that 2×RBD27 can be used as a specific tool to inhibit Rab27 function(s) in melanocytes.

### The inhibitory effect of 2×RBD27 on melanosome transport is rapidly neutralized by AID technology

**Fig. 3.**
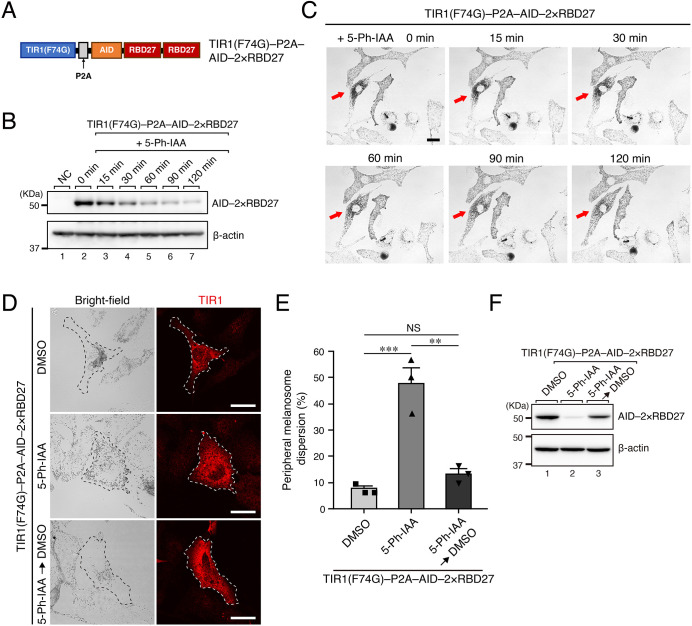
**Perinuclear melanosome aggregation induced by AID–2×RBD27 in melan-a cells was restored after its rapid degradation by auxin-inducible degron technology.** (A) Schematic representation of TIR1(F74G)–P2A–AID–2×RBD27. (B) Degradation of AID–2×RBD27 in melan-a cells after the addition of 1 μM 5-phenyl-indole-3-acetic acid (5-Ph-IAA), as revealed by immunoblotting with the antibodies indicated. NC, negative control (untransfected cells). (C) Time-dependent dispersion of melanosomes to the cell edge in AID–2×RBD27-expressing melan-a cells after the addition of 5-Ph-IAA. Red arrows point to cells expressing AID–2×RBD27. Scale bar: 20 μm. (D) Typical images of melan-a cells expressing AID–2×RBD27 under control conditions (DMSO; top row), 5-Ph-IAA-treated conditions (middle row) and 5-Ph-IAA-washout conditions (bottom row), achieved by incubation for an additional 5 h in fresh medium containing DMSO (without 5-Ph-IAA). Transfected cells were identified by immunostaining for TIR1 (outlined with dashed lines), and corresponding bright-field images are shown. Scale bars: 20 μm. (E) The percentages of cells exhibiting peripheral melanosome dispersion in D. Data represent the means±s.e.m. of data obtained in three independent experiments (*n*=30 cells in each experiment). NS, not significant; **P<0.01; ***P<0.001 (one way ANOVA and Tukey's test). (F) Expression of AID–2×RBD27 in melan-a cells under control conditions, 5-Ph-IAA-treated conditions and 5-Ph-IAA-washout conditions, as revealed by immunoblotting with the antibodies indicated. The positions of the molecular mass markers (kDa) are shown on the left in B and F. Images in B and F are representative of two experimental repeats, and those in C of ten experimental repeats.

Because expression of 2×RBD27 in melanocytes strongly induced perinuclear melanosome aggregation (Fig. 2C), inactivation or depletion of 2×RBD27 should disperse melanosomes from the perinucleus to the cell edge and allow us to specifically observe the anterograde movements of melanosomes by live-cell imaging. We decided to use the recently developed AID technology (Yesbolatova et al., 2020) to rapidly degrade the 2×RBD27 that had been expressed in the melanocytes, and prepared a vector that can simultaneously express AID-tagged 2×RBD27 and a ubiquitin ligase complex component, TIR1(F74G), separated by the P2A self-cleavage site [named TIR1(F74G)–P2A–AID–2×RBD27; Fig. 3A] (Kim et al., 2011). We then transfected melan-a cells with this vector and evaluated the degradation of AID–2×RBD27 after treatment with 1 μM 5-phenyl-indole-3-acetic acid (5-Ph-IAA; i.e. the auxin analog) by immunoblotting. The results showed that degradation of AID–2×RBD27 progressed after 5-Ph-IAA treatment and that more than 80% of the AID–2×RBD27 had disappeared 120 min after treatment, although the AID–2×RBD27 band was still visible (Fig. 3B). Next, after the addition of 5-Ph-IAA, we used live-cell imaging to observe the movements of the melanosomes in melan-a cells that expressed TIR1(F74G)–P2A–AID–2×RBD27 (referred to as AID–2×RBD27, unless otherwise specified) and exhibited perinuclear melanosome aggregation. As shown in Fig. 3C, the melanosomes began to gradually disperse 30 min after the addition of 5-Ph-IAA, and they reached the cell edge by 60–120 min. At 5 h after the addition of 5-Ph-IAA or DMSO (control), we calculated the percentage of cells exhibiting peripheral melanosome dispersion in the TIR1(F74G)-positive cells (Fig. 3D, top and middle rows). The results showed that ∼50% of the 5-Ph-IAA-treated cells exhibited peripheral melanosome dispersion compared to only ∼10% of the control DMSO-treated cells (Fig. 3E). The incomplete rescue effect of 5-Ph-IAA on peripheral melanosome distribution is presumably caused by the presence of undegraded AID–2×RBD27 (see Fig. 3B, lane 7), which still inhibits the function of Rab27A in melanosome transport. We then investigated whether the inhibitory effect of AID–2×RBD27 would reverse under 5-Ph-IAA-washout conditions. After adding 5-Ph-IAA and letting the cells stand for 5 h, we incubated them for an additional 5 h in fresh medium containing DMSO alone. As expected, melan-a cells expressing AID–2×RBD27 once again exhibited perinuclear melanosome aggregation, and the percentage of melanosome-dispersed cells had decreased to the same level as that in the DMSO-treated cells (Fig. 3D, bottom row; Fig. 3E). Immunoblotting confirmed the degradation of AID–2×RBD27 after the addition of 5-Ph-IAA and its restoration after 5-Ph-IAA washout (Fig. 3F).

Then, to determine whether expression of AID–2×RBD27 or TIR1(F74G) affects the structure of the cytoskeleton itself, we stained cytoskeletal structures (i.e. microtubules and actin filaments) in both AID–2×RBD27-expressing cells and control cells (Fig. S1, top and bottom rows). In contrast to the nocodazole-treated cells (with inhibition of microtubule polymerization) or cytochalasin D-treated cells (with inhibition of actin polymerization), the cytoskeletal structures of the AID–2×RBD27-expressing cells appeared normal, the same as in the control cells. Taken together, these results indicated that AID–2×RBD27 can serve as a tool to reversibly inhibit the function of Rab27 without affecting cytoskeletal structures.

### Kinetic analyses of anterograde melanosome transport from the perinucleus to the cell edge in AID–2×RBD27-expressing melan-a cells

**Fig. 4.**
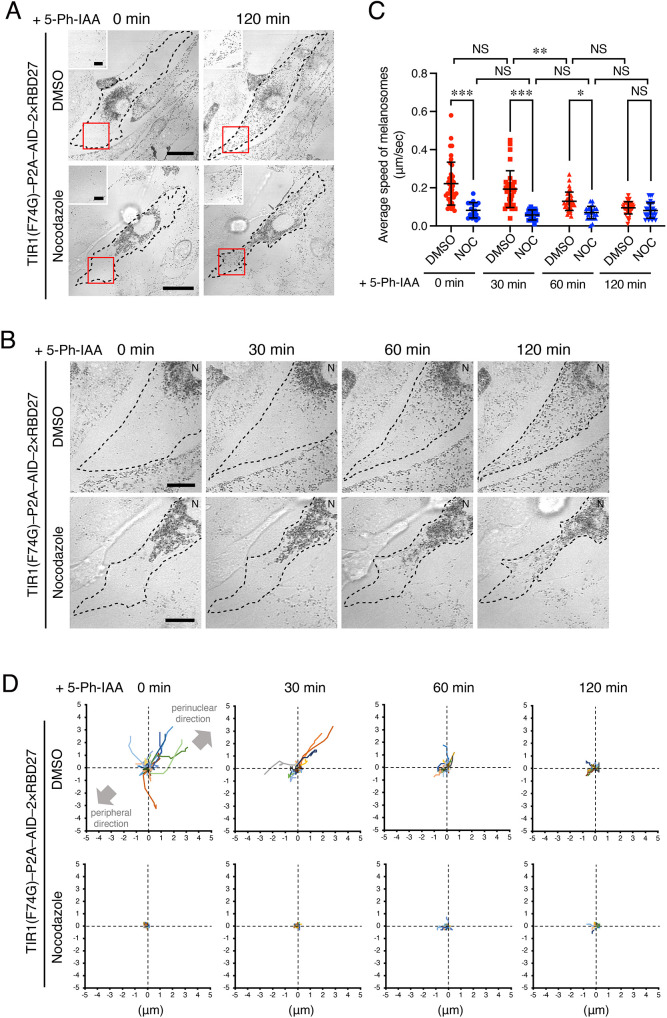
**Kinetic analyses of 2D melanosome transport after the addition of 5-Ph-IAA to melan-a cells expressing AID–2×RBD27.** (A) Typical images 0 min or 120 min after the addition of 5-Ph-IAA to control (i.e. DMSO-treated) and 10 μM nocodazole-treated AID–2×RBD27-expressing melan-a cells. Insets show magnified views of the boxed areas. Scale bars: 20 μm (5 µm in insets). (B) Enlarged images of A, showing the distribution of melanosomes over time after the addition of 5-Ph-IAA. Scale bars: 20 μm. N, nucleus. Cells in A and B are outlined by dashed lines. Images in A and B are representative of three experimental repeats. (C) Average speed of melanosomes at the times indicated after the addition of 5-Ph-IAA to control and nocodazole (NOC)-treated AID–2×RBD27-expressing melan-a cells. More than 20 melanosomes were randomly selected from one experiment, and their speed was determined with the manual tracking plugin of ImageJ software. Data represent the means±s.d. NS, not significant; *P<0.05; **P<0.01; ***P<0.001 (two-way ANOVA and Bonferroni test). (D) Trajectory analysis of melanosomes corresponding to C. The trajectory of each melanosome movement was drawn for 10 s, with 0 μm as the base point.

We next used the AID–2×RBD27 system established above to analyze anterograde melanosome transport, especially microtubule-dependent anterograde melanosome transport, the importance of which is not fully understood (Wu et al., 1998; Evans et al., 2014; Ishida et al., 2015; Oberhofer et al., 2017; Robinson et al., 2017; Subramaniam et al., 2021 preprint). We pretreated AID–2×RBD27-expressing cells with DMSO or nocodazole and, after adding 5-Ph-IAA, performed 2D live-cell imaging for 120 min under a light microscope (Fig. 4A,B). Although melanosomes in both the control DMSO-treated and nocodazole-treated cells gradually began to disperse after 30 min and reached the cell edge by 120 min (Fig. 4B), kinetic analyses of each melanosome movement revealed the differences in the melanosome movements in terms of speed and distance migrated. In the control cells, fast-moving melanosomes (>0.3 μm/s) were often observed at 0 min and 30 min after the addition of 5-Ph-IAA, but the average speed of the melanosome movements significantly slowed at 60 min and remained slow until 120 min (Fig. 4C, red symbols). We also tracked the 10-s trajectories of the melanosome movements that had been analyzed in Fig. 4C (red symbols). The results showed that several melanosomes with long migratory distances (>3 μm during 10 s) in the anterograde direction were observed at 0 min and 30 min (although retrograde movements were also observed), whereas most melanosomes had short migratory distances (<3 μm during 10 s) at 60 min and 120 min (Fig. 4D, top row). In the nocodazole-treated cells, however, melanosome movement speed was significantly slower than that in the control cells at 0 min, 30 min and 60 min after the addition of 5-Ph-IAA, and it remained slow until 120 min (Fig. 4C, blue symbols). Moreover, the melanosomes in the nocodazole-treated cells always migrated shorter distances (<3 μm during 10 s) than those in the control cells, and their migratory distances were almost constant until 120 min (Fig. 4D, bottom panels). These results indicated that the fast, long-range melanosome movements observed only in the early stage of the recovery of peripheral melanosome distribution (0 min and 30 min) are likely to be attributable to microtubule-dependent transport and the slow, intermittent melanosome movements are likely to be attributable to actin-dependent transport.

### Three-dimensional live-cell imaging of two distinct speeds of anterograde melanosome transport in melanocytes

**Fig. 5.**
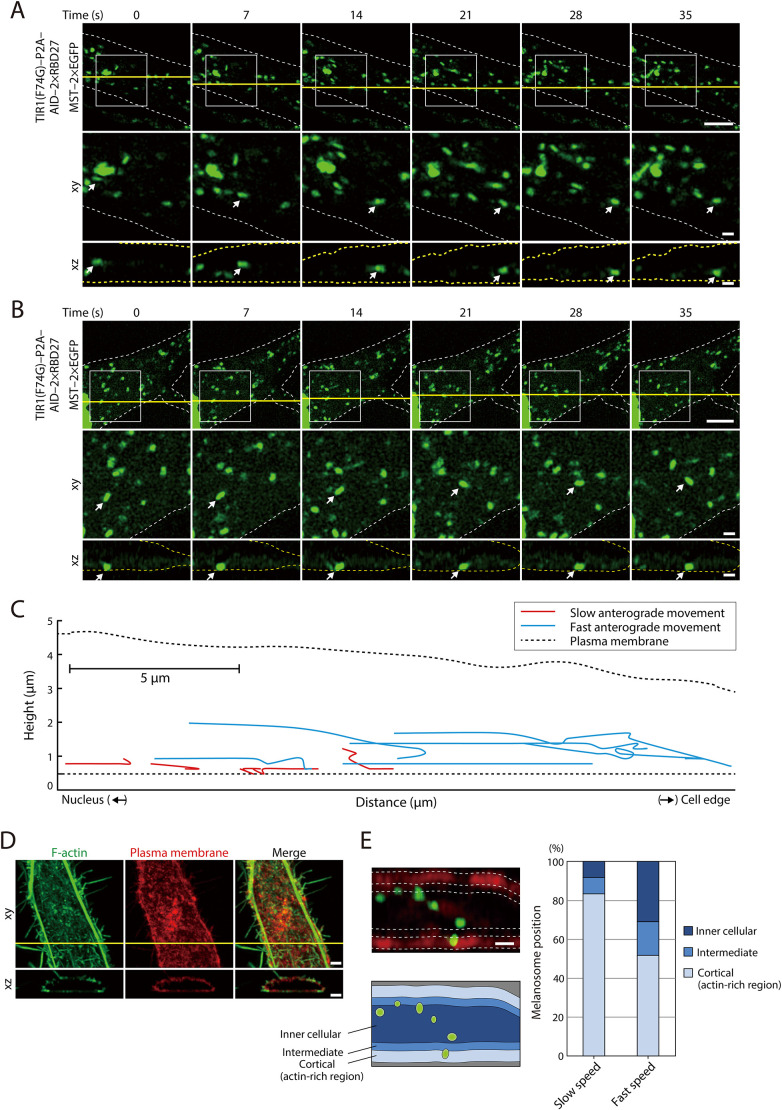
**3D live-cell imaging of two distinct speeds of melanosome transport after the addition of 5-Ph-IAA to melan-a cells expressing AID–2×RBD27.** (A,B) Live-cell imaging after the addition of 5-Ph-IAA to melan-a cells expressing AID–2×RBD27 and MST–2×EGFP (melanosome marker; green). The images were stacked by *z*-projection. The arrows point to tracked fast-moving melanosomes (A) and slow-moving melanosomes (B). The horizonal sections of the *z*-stack images (yellow lines in the top rows) are shown in the bottom rows. Dashed lines outline the cells that were predicted by plasma membrane staining with PlasMem Bright Red after live-cell imaging. Scale bars: 5 μm (1 μm in magnified views). Images in A and B are representative of seven experimental repeats. (C) Examples of fast and slow anterograde melanosome movements. The trajectory of each melanosome movement was drawn at 7 s intervals until a maximum of 70 s. (D) Typical *z*-stack images of melan-a cells. The horizonal sections of the *z*-stack images (yellow lines in the top row) are shown in the bottom row. The cells were stained for F-actin (phalloidin, green) and with PlasMem Bright Red (plasma membrane, red). Scale bars: 2 μm. Images representative of four experimental repeats. (E) Definitions of the regions in which melanosomes were located in the melanocytes. The cortical region (actin-rich region), intermediate region and inner cellular region are defined as the region stained with PlasMem Bright Red (plasma membrane, red; ∼12.5% of the peripheral area; see D), the region that accounts for 12.5–25% of the inner area that lies directly below the cortical region and the rest of the inner region, respectively. Scale bars: 1 μm. The graph on the right shows the percentages of melanosomes located in each region. We defined more than 0.3 μm/s melanosome transport as fast speed and the rest as slow speed. Under our experimental conditions, most of the melanosome movements occurred at the bottom of the cell, presumably because the microtubule-organizing center, where melanosomes are mainly aggregated, is located at the bottom of the cell (see Fig. S4). Slow melanosome movements (*n*=36) and fast melanosome movements (*n*=29) were obtained from seven independent live-cell imaging data.

In general, the spatial distributions of microtubules and actin filaments are quite different, with microtubules being present in the inner cellular region and actin filaments in the cortical region. We confirmed the different spatial distributions of microtubules and actin filaments in melanocytes by 3D fluorescence examination (Fig. S2A,B). We then attempted to determine whether the fast melanosome movements described above occur in the microtubule-enriched inner cellular region and slow melanosome movements occur in the cortical actin-enriched region. We did so by expressing AID–2×RBD27 together with melanosome-targeting tag (MST)–2×EGFP, a known melanosome marker (Ishida et al., 2014), in melan-a cells and performing 3D live-cell imaging after the addition of 5-Ph-IAA (Fig. 5A–C). The results of a tracking analysis of individual melanosomes in an *x*–*z* plane indicated that fast-moving melanosomes often passed through the central region of the cell (Fig. 5A, bottom row; Fig. 5C, blue lines), whereas slow-moving melanosomes passed near the plasma membrane (Fig. 5B, bottom row; Fig. 5C, red lines). To quantitatively analyze melanosome movements in 3D, we then divided the cell into three regions – an inner cellular region, in which most microtubules are present; an intermediate region; and a cortical region, in which most actin filaments are present (see Fig. 5E and its legend for details) – and analyzed intracellular melanosome positions according to melanosome movement speed (Fig. 5D,E, left column). The results showed that more than 80% of the slow-moving melanosomes were located in the cortical region, whereas ∼50% of the fast-moving melanosomes were located in the inner cellular and intermediate regions (Fig. 5E, right).

### Live-cell imaging of anterograde melanosome transport in dendritic melanocytes

**Fig. 6.**
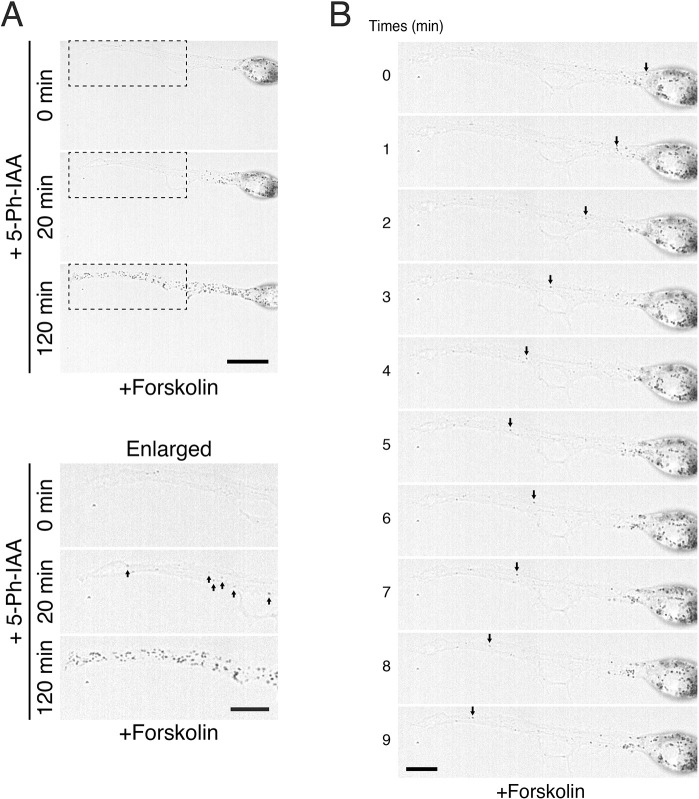
**Live-cell imaging of anterograde melanosome transport after the addition of 5-Ph-IAA to dendritic melan-a cells expressing AID–2×RBD27.** (A) Typical images of AID–2×RBD27-expressing dendritic melan-a cells at 0 min, 20 min and 120 min after the addition of 5-Ph-IAA. The bottom three panels are enlarged images of the areas outlined by dashed line boxes in the top three panels. The arrows point to fast-moving melanosomes, likely occurring along microtubules. Scale bars: 20 μm (10 μm in enlarged images). (B) Live-cell imaging of the dendrite after the addition of 5-Ph-IAA to melan-a cells expressing AID–2×RBD27. The arrows point to tracked fast-moving melanosomes (see also Movies 1 and 2). Scale bar: 10 μm. Images in A and B are representative of three experimental repeats.

Lastly, because epidermal melanocytes are known to extend microtubule-enriched dendrites and contact many surrounding keratinocytes via their dendrites in skin tissues (Kippenberger et al., 1998), we used AID–2×RBD27 to investigate whether fast, long-range melanosome transport along microtubules contributes to the dendritic localization of melanosomes. We did this by treating melan-a cells expressing AID–2×RBD27 with forskolin, an adenylate cyclase activator, to promote the formation of dendrites (Passeron et al., 2004; Ohbayashi et al., 2012), which contain abundant microtubules in their central region (Fig. S2C). As shown in Fig. 6A (top panel), in the absence of 5-Ph-IAA, the melanosomes were aggregated in the cell body, and only a few melanosomes were observed in the dendrites. However, 20 min after 5-Ph-IAA treatment, several melanosomes had been rapidly transported to the distal part of the dendrites (Fig. 6A, middle panel), and they had been transported at a fast movement speed, likely along microtubules (Fig. 6A, arrows in the middle panel in the enlarged images; Fig. 6B; Movie 1). As in the melanocytes without dendrites (Fig. 4), 120 min after the addition of 5-Ph-IAA, the melanosomes were distributed in the peripheral region, including at the tip of the dendrite (Fig. 6A, bottom panel), and slow-moving melanosomes along the cortical actin in the dendrite appeared to be dominant at a later stage of melanosome dispersion (20–120 min after the addition of 5-Ph-IAA; Movie 2).

## DISCUSSION

**Fig. 7.**
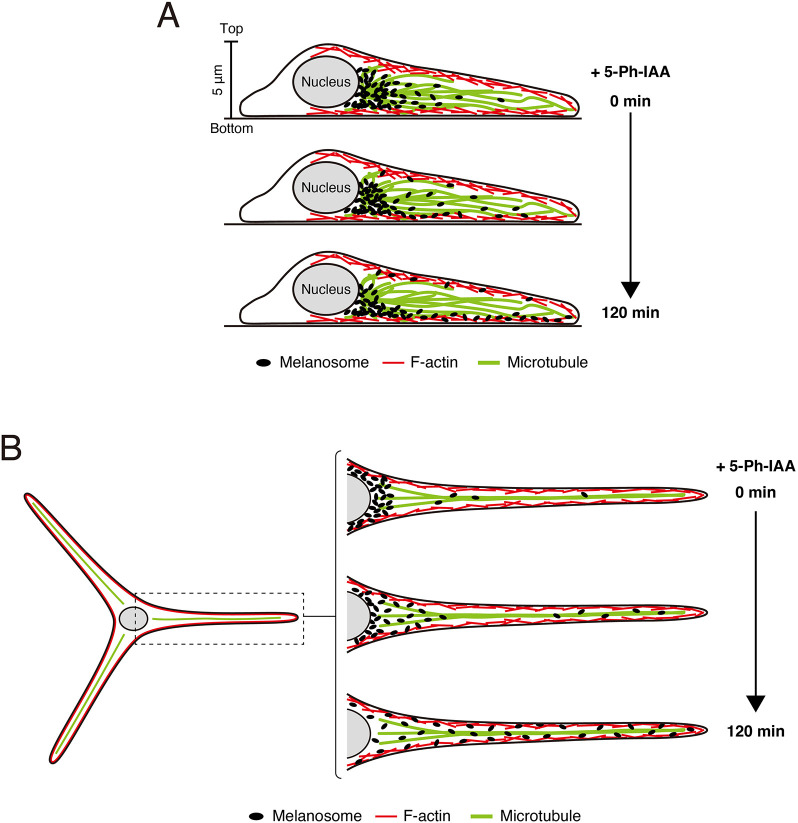
**Working models of anterograde melanosome transport in the process of melanosome dispersion to the cell edge of cultured melanocytes with and without dendrites.** (A) Melanosome dispersion in a melanocyte without dendrites. Once degradation of AID–2×RBD27 starts, in response to 5-Ph-IAA, some of the perinuclear melanosomes are rapidly transported along microtubules at a long range in the inner cellular region, then are immediately trapped by cortical actin and transported to the edge of the cell. By contrast, the majority of the melanosomes are slowly and intermittently transported to the edge of the cell along cortical actin filaments. (B) Melanosome dispersion in the dendritic melanocyte. Rapid, long-range transport of several melanosomes along microtubules in the dendrite occurs initially, while other melanosomes are slowly and intermittently transported (occasionally exhibit back and forth movements) to the dendrite along cortical actin filaments.

In the present study, we developed a novel tool that reversibly inhibits the function of Rab small GTPases by using AID technology, which induces rapid degradation of an AID-tagged Rab effector domain after the addition of an auxin analog. We demonstrated the usefulness of AID–2×RBD27 in Rab27A-mediated melanosome transport in melanocytes as a model. We succeeded in showing that expression of AID–2×RBD27 in melanocytes induces melanosome aggregation to the perinucleus, a typical Rab27A-inhibition phenotype, and that redispersion to the cell edge occurs in response to 5-Ph-IAA (Fig. 3C). By using the AID–2×RBD27 system in combination with 3D live-cell imaging, we found that two types of anterograde melanosome movements occur during recovery of peripheral melanosome dispersion: fast, long-range melanosome movements in the microtubule-enriched inner cellular region only in the early stage of the recovery and slow, intermittent melanosome movements in the actin-enriched cortical region, especially in the late stage (Figs 4 and 5). Based on these findings, we proposed a model in which some melanosomes are first transported in a microtubule-dependent manner and then by actin-dependent transport, with the majority of the melanosomes being transported to the cell edge along the cortical actin filaments (Fig. 7A). The former fast, long-range melanosome movements along microtubules appeared to be important for rapid, efficient localization of melanosomes at the tip of dendrites (Fig. 6 and Fig. 7B). Further research will be necessary to evaluate the importance of anterograde melanosome transport along microtubules in skin pigmentation using epidermal melanocytes that extend microtubule-enriched dendrites three dimensionally in skin tissues.

Melanosomes reached the edge of the cell even in the presence of nocodazole, which collapsed microtubules (Fig. 4A,B), indicating that microtubule-dependent movements are less important than actin-dependent movements in anterograde melanosome transport, as has been reported previously (Evans et al., 2014). A limited contribution by microtubule-dependent anterograde melanosome transport has also been reported in a previous study that demonstrated that depletion of the Rab1A–SKIP (also known as PLEKHM2)–kinesin-1 (Kif5b) complex, which functions in anterograde melanosome transport, caused inhibition of peripheral melanosome distribution in only 30% of the depleted cells (Ishida et al., 2015). We speculate that the limited contribution of this complex to anterograde melanosome transport is attributable to the limited number of the functional melanosomal receptor Rab1A, which is mainly localized at the Golgi complex and regulates endoplasmic reticulum–Golgi trafficking (Allan et al., 2000). Consistent with our speculation, forced recruitment of Kif5b on melanosomes by using Kif5bΔCC–EGFP–SHD, which contains the Rab27A-binding domain instead of the tail domain of Kif5b, was able to rescue the melanosome aggregation phenotype of melan-ln (another immortal mouse melanocyte cell line) cells genetically lacking the Rab27A effector melanophilin (also known as Slac2-a) (Hume et al., 2006). As shown in Fig. S3, the melanosomes in Kif5bΔCC–EGFP–SHD-expressing cells seemed to have hyper-accumulated at the cell edge, whereas the control Kif5bΔCC–EGFP-expressing cells showed no effect on perinuclear melanosome aggregation. Thus, under increased Kif5b–melanosomal receptor conditions, we were able to observe microtubule-dependent melanosome movements to the cell edge more clearly.

We propose that two important factors, melanosome size and cell thickness, contribute to the process of supporting melanosome transport to the cell edge along the cortical actin filaments, especially in nocodazole-treated cells. The maximum thickness of cultured melanocytes is ∼5 μm (Fig. 5C), and the size of their melanosomes is relatively larger than that of other organelles (>0.5 μm). Because the distance from the nucleus is greater, the cells are thinner, thereby increasing the possibility of melanosome trapping by cortical actin. Once melanosomes are trapped by cortical actin, they are slowly and intermittently transported to the cell edge along the cortical actin filaments.

The AID–2×RBD27 established in this study requires only a specific Rab effector domain and not Rab27A itself; therefore, the AID–RBD (or AID–2×RBD) system will be easy applied to other Rabs as long as their specific Rab effector domains (e.g. RBD11; Osaki et al., 2021) are available and can be used as a temporary, reversible inhibitor of specific membrane trafficking events in the future. In addition, because AID–2×RBD27 and TIR1(F74G), which are linked by the P2A self-cleavage site (Fig. 3A), can be easily co-expressed in a single cell, this system would be useful in conducting inhibition–rescue experiments by performing a simple plasmid transfection.

In conclusion, we have developed an AID–2×RBD27 system that reversibly inhibits the function of Rab27 and have succeeded in three dimensionally observing anterograde melanosome dispersion from the nucleus to the cell edge in melanocytes. Application of this system should provide new insights into the mechanisms of dynamic membrane trafficking events that are regulated by Rab small GTPases.

## MATERIALS AND METHODS

### Materials

The antibodies, plasmids and primers used in this study are summarized in Table S1. Unless otherwise specified, all other materials used in this study were analytical grade or the highest grade commercially available. The following plasmids used in this study [pEGFP-C1-2×RBD27-WT (or -mut), pEF-TIR1(F74G)-P2A-AID-2×RBD27, pEF-MST-2×EGFP, pEF-Kif5b-ΔCC-EGFP and pEF-Kif5b-ΔCC-EGFP-SHD] are available from the RIKEN BioResource Research Center in Japan (https://dnaconda.riken.jp/search/depositor/dep005893.html; RDB20906–RDB20911).

### Molecular cloning and plasmid construction

cDNAs of the N-terminal 87 amino acids of rat Slp2-a and mouse Slp2-a (RBD27-WT) were amplified from cDNAs of rat PC12 cells and the Marathon-Ready mouse brain cDNAs (Takara Bio, Shiga, Japan), respectively, by PCR using the specific pairs of oligonucleotides shown in Table S1. Rat RBD27 and mouse RBD27 were then arranged in tandem (Fig. 2A) by standard molecular biology techniques. cDNAs of AID and *Oryza sativa* TIR1 were prepared as described previously (Nishimura et al., 2009; Hatoyama et al., 2021). A point mutant of mouse RBD27 (E11A/R32A; named RBD27-mut) was also prepared as described previously (Kuroda and Fukuda, 2004). Rat RBD27-mut and a TIR1(F74G) mutant (Yesbolatova et al., 2020) were prepared by standard molecular biology techniques, using oligonucleotides shown in Table S1. The 2×RBD27 (i.e. tandem RBD27) cDNA was prepared by connecting rat and mouse RBD27 with a glycine linker (GGSGGTGGS). These cDNAs were subcloned into the appropriate expression vectors shown in Table S1. To simultaneously express TIR1(F74G) and AID-tagged 2×RBD27 in a single cell, they were connected via the P2A self-cleavage site (ATNFSLLKQAGDVEENPGP; see Fig. 3A) (Kim et al., 2011), and their corresponding cDNA was subcloned into the modified pEF-BOS expression vector. MST-2×EGFP cDNA (Ishida et al., 2014) was also prepared by standard molecular biology techniques and subcloned into the modified pEF-BOS expression vector.

### Yeast two-hybrid assays

The yeast strain, medium, culture conditions and transformation protocol used were as described previously (James et al., 1996). The yeast two-hybrid assays were performed using pGBD-C1-Rabs(CA)ΔCys [CA, constitutively active; ΔCys, lacking a C-terminal cysteine residue(s) for geranylgeranylation] and pAct2-RBD27 (WT or mutant) as described previously (Ohishi et al., 2019). Yeast cells on the selection medium (synthetic complete medium lacking adenine, histidine, leucine and tryptophan, made in house) were incubated at 30°C for ∼1 week. Data shown are representative of the data obtained in two independent experiments, and similar results were obtained in each experiment.

### Cell cultures and transfection

Melan-a cells and melan-ln cells, immortal mouse melanocyte cell lines derived from a black mouse and *leaden* mouse [i.e. melanophilin-deficient mouse], respectively, were obtained from the Wellcome Trust Functional Genomics Cell Bank at St George's, University of London, and cultured as described previously (Bennett et al., 1987; Kuroda et al., 2003; Hume et al., 2006). COS-7 cells were cultured at 37°C in Dulbecco's modified Eagle medium (FUJIFILM Wako Pure Chemical, Osaka, Japan) supplemented with 10% fetal bovine serum (MP Biomedicals, Irvine, CA), 100 U/ml penicillin and 100 μg/ml streptomycin, in a 5% CO_2_ incubator. Cells were transfected with plasmid DNAs using Lipofectamine 2000 or Lipofectamine 3000 (Thermo Fisher Scientific, Waltham, MA), according to the manufacturer's instructions.

### Co-immunoprecipitation assays and direct binding assays

For the co-immunoprecipitation assays in COS-7 cells, cells that had been transfected for 2 days with pEF-T7-RBD27 (WT or mutant) or pEF-FLAG-Rab27A were lysed with a lysis buffer [50 mM HEPES-KOH, pH 7.2, 250 mM NaCl, 1 mM MgCl_2_, 1% Triton X-100, 0.5 mM GTPγS and cOmplete™, EDTA-free protease inhibitor cocktail (Roche, Basel, Switzerland)]. The cell lysates containing T7–RBD27 (WT or mutant) or FLAG–Rab27A were incubated for 1 h at 4°C with anti-T7 tag antibody-conjugated agarose beads (Novagen-Merck, Darmstadt, Germany). After washing the beads three times with a washing buffer (50 mM HEPES-KOH, pH 7.2, 150 mM NaCl, 1 mM MgCl_2_ and 0.1% Triton X-100), proteins bound to the beads were analyzed by 15% SDS-PAGE followed by immunoblotting with the appropriate horseradish peroxidase (HRP)-conjugated antibodies listed in Table S1. Immunoreactive bands were visualized by enhanced chemiluminescence, and images were captured with a ChemiDoc Touch Imaging System (Bio-Rad, Hercules, CA).

For the direct binding assays, T7–GST–RBD27 expressed in COS-7 cells and GST–Rab27A expressed in bacteria were affinity purified with anti-T7 tag antibody-conjugated agarose beads and glutathione Sepharose^TM^ 4B (Cytiva, Marlborough, MA), respectively. To remove unrelated proteins in COS-7 cell lysates from anti-T7 tag antibody-conjugated agarose beads, the beads were washed three times with a 250 mM NaCl-containing buffer (50 mM HEPES-KOH, pH 7.2, 1 mM MgCl_2_ and 0.1% Triton X-100), and the purity of T7-tagged proteins was verified both by Coomassie Brilliant Blue (CBB) staining and by immunoblotting (see also Fig. S5) with anti-T7 tag antibody (data not shown). The beads coupled with T7–GST–RBD27 were incubated for 1 h at 4°C with 10 μg purified GST–Rab27A or GST alone as a control in 50 mM HEPES-KOH, pH 7.2, 150 mM NaCl, 1 mM MgCl_2_, 0.5 mM GTPγS and 0.1% Triton X-100. After washing the beads with the washing buffer three times, proteins bound to the beads were analyzed by 12.5% SDS-PAGE followed by CBB staining. The original immunoblot data and stained gels used in this study are shown in Fig. S5.

### Melanosome distribution assays

Three days after transfecting plasmid DNAs (final concentration, 1 μg/ml) into melan-a cells, the percentage of cells exhibiting perinuclear melanosome aggregation or dispersion was calculated after a manual cell count. Cells in which more than 50% of the melanosomes were present around the nucleus were classified as ‘aggregated’ (Kuroda et al., 2003), and the remaining cells were classified as ‘dispersed’ (Fig. 2C and Fig. 3E).

Three days after transfecting plasmid DNAs (final concentration, 1 μg/ml) into melan-ln cells, the percentage of cells exhibiting peripheral melanosome accumulation was calculated after a manual cell count. Cells in which more than 50% of the melanosomes were present at the periphery of the cells were classified as ‘peripheral accumulation’ (Ishida et al., 2015) (Fig. S3B).

### Immunofluorescence analysis

Three days after transfecting pEF-TIR1(F74G)-P2A-AID-2×RBD27 into melan-a cells, the cells were fixed with 4% paraformaldehyde for 10 min, permeabilized with 0.05% saponin for 30 min and blocked with 1% bovine serum albumin in PBS for 30 min. The cells were then stained for 1 h with anti-TIR1 antibody (1:200 dilution; PD048, MBL, Nagoya, Japan; RRID, AB_2909494) and anti-α-tubulin antibody (1:100 dilution; T6199, Sigma-Aldrich, St. Louis, MA; RRID, AB_477583) and visualized with an appropriate Alexa Fluor-conjugated secondary antibody. For F-actin staining, Alexa Fluor-conjugated phalloidin (Invitrogen, Thermo Fisher Scientific) was used (1:1000 dilution; 1 h). For plasmid transfection into melan-ln cells, the cells were fixed with 4% paraformaldehyde for 10 min, permeabilized with 0.05% saponin for 30 min and blocked with 1% bovine serum albumin in PBS for 30 min. The cells were then stained for 1 h with anti-GFP antibody (1:2000 dilution; 598, MBL; RRID, AB_591816) and visualized with an appropriate Alexa Fluor-conjugated secondary antibody listed in Table S1. The stained cells were examined for fluorescence with a confocal fluorescence microscope (FluoView 1000-D, Evident/Olympus, Tokyo, Japan) through an objective lens (60× magnification; numerical aperture, 1.40; Evident/Olympus) and with FluoView software (version 4.1a; Evident/Olympus). The images were processed with ImageJ software (version 2.1.0/1.53c; National Institutes of Health).

### Live-cell imaging of peripheral melanosome dispersion using AID technology

For confocal fluorescence microscopy, 3 days after transfecting pEF-TIR1(F74G)-P2A-AID-2×RBD27 into melan-a cells, the cells were placed in a 10% CO_2_ culture chamber (MI-IBC, Evident/Olympus) maintained at 37°C, and live-cell imaging was performed after the addition of 5-Ph-IAA (BioAcademia, Osaka, Japan; final concentration, 1 μM). For live imaging of melan-a cells with disrupted microtubules, cells were preincubated for 1 h with 10 μM nocodazole, and 5-Ph-IAA was added before the start of live imaging. A 100× objective lens (numerical aperture, 1.40; Evident/Olympus) was used for live-cell imaging, and bright-field images of cells were captured at 1 s intervals for 120 min. A 100× objective lens was used to acquire *z*-stack images, and the appropriate number of images was taken at 0.1 μm intervals in the *z*-axis direction. In some cases, the cells that had been used for live-cell imaging were further immunostained with anti-TIR1 antibody to confirm that TIR1(F74G)–P2A–AID–2×RBD27 was actually expressed in them (data not shown). For live imaging of dendritic melan-a cells, cells expressing AID–2×RBD27 were first treated with 20 μM forskolin for 20 h and then 5-Ph-IAA was added, as described above.

### 3D live-cell imaging

Three days after transfecting pEF-TIR1(F74G)-P2A-AID-2×RBD27 and pEF-MST-2×EGFP into melan-a cells, the cells were placed in a 10% CO_2_ culture chamber (STRG-WELSX-SET, Tokai Hit, Shizuoka, Japan) maintained at 37°C for imaging with an Andor Dragonfly spinning disk scanning unit (Dragonfly200, Oxford Instruments, Abingdon, UK), and live-cell imaging was performed after the addition of 5-Ph-IAA. A 60× objective lens (numerical aperture, 1.35; Evident/Olympus) was used for live-cell imaging, and fluorescence images of cells were captured at 7 s intervals for 15 min. Outlines of the cells were predicted by plasma membrane staining with PlasMem Bright Red (P505, DOJINDO Laboratories, Kumamoto, Japan) after live-cell imaging. To acquire *z*-stack images, the appropriate number of images was taken at 0.15 μm intervals in the *z*-axis direction.

### Kinetic analyses of melanosome transport

Images acquired by live-cell imaging were analyzed for the speed of melanosome movements using the manual tracking plugin of ImageJ software. Melanosome migration distances were analyzed for the trajectory of melanosome movements using the manual tracking plugin of ImageJ software, and these data were plotted using the DiPer program Plot_At_Origin for Microsoft Excel (Gorelik and Gautreau, 2014).

### Statistical analysis

Statistical analysis was performed by one-way ANOVA followed by Tukey's test or Dunnett's test (for multiple comparisons) and by two-way ANOVA followed by Bonferroni test or unpaired two-tailed Student's *t*-test (for comparisons between two samples), using GraphPad Prism 9 software (GraphPad Software, San Diego, CA). All quantitative data are expressed as the means±s.d. or s.e.m. *P*<0.05 was considered significant.

## Supplementary Material



10.1242/joces.263878_sup1 Supplementary information
