# Supplementary material for: AID–2×RBD27, an auxin-inducible degron-based Rab27 trapper that reversibly inhibits the function of Rab27A in melanocytes
Source: J Cell Sci. 2025 Jun 10;138(11):jcs263878. doi: 10.1242/jcs.263878 (PMC12188314; doi:10.1242/jcs.263878)
Supplement: Supplementary information [file joces-138-263878-s1.pdf]

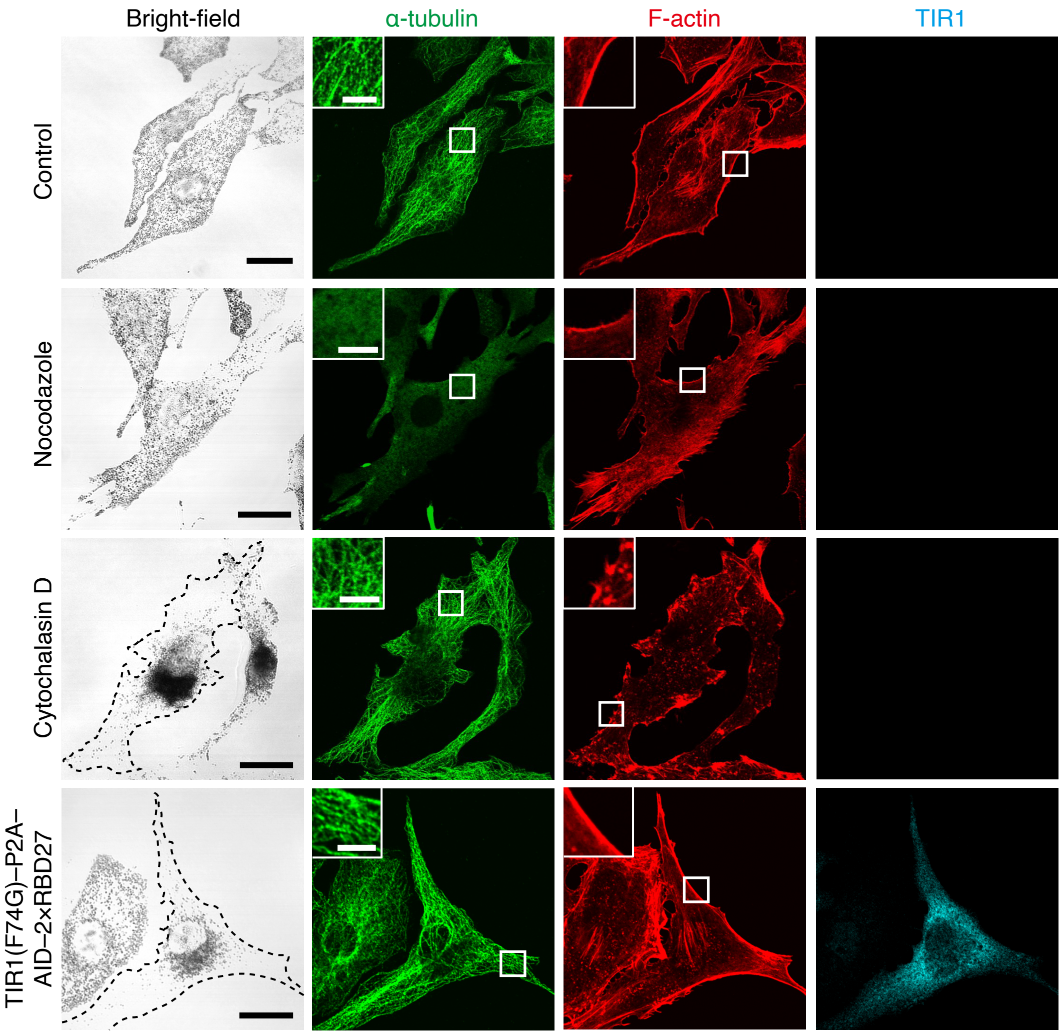

Fig. S1. Expression of RBD27 or TIR1(F74G) had no effect on cytoskeletal structures in melan-a cells. Typical images of cytoskeletal structures of control and TIR1(F74G)–P2A–AID–2×RBD27-expressing melan-a cells. The cells were stained for α-tubulin (green), F-actin (phalloidin, red), and TIR1 (cyan). Melan-a cells were exposed to 10 µM nocodazole for 1 h (second row of panels) and to 2 µM cytochalasin D treatment for 1 h (third row of panels) to confirm the disruption of microtubules and actin filaments, respectively. The insets show magnified views of the boxed areas. Scale bars, 20 µm. (insets) 5 µm. Cells exhibiting perinuclear melanosome aggregation are outlined with broken lines (third from the top and bottom bright-field image panels).

Journal of Cell Science • Supplementary information

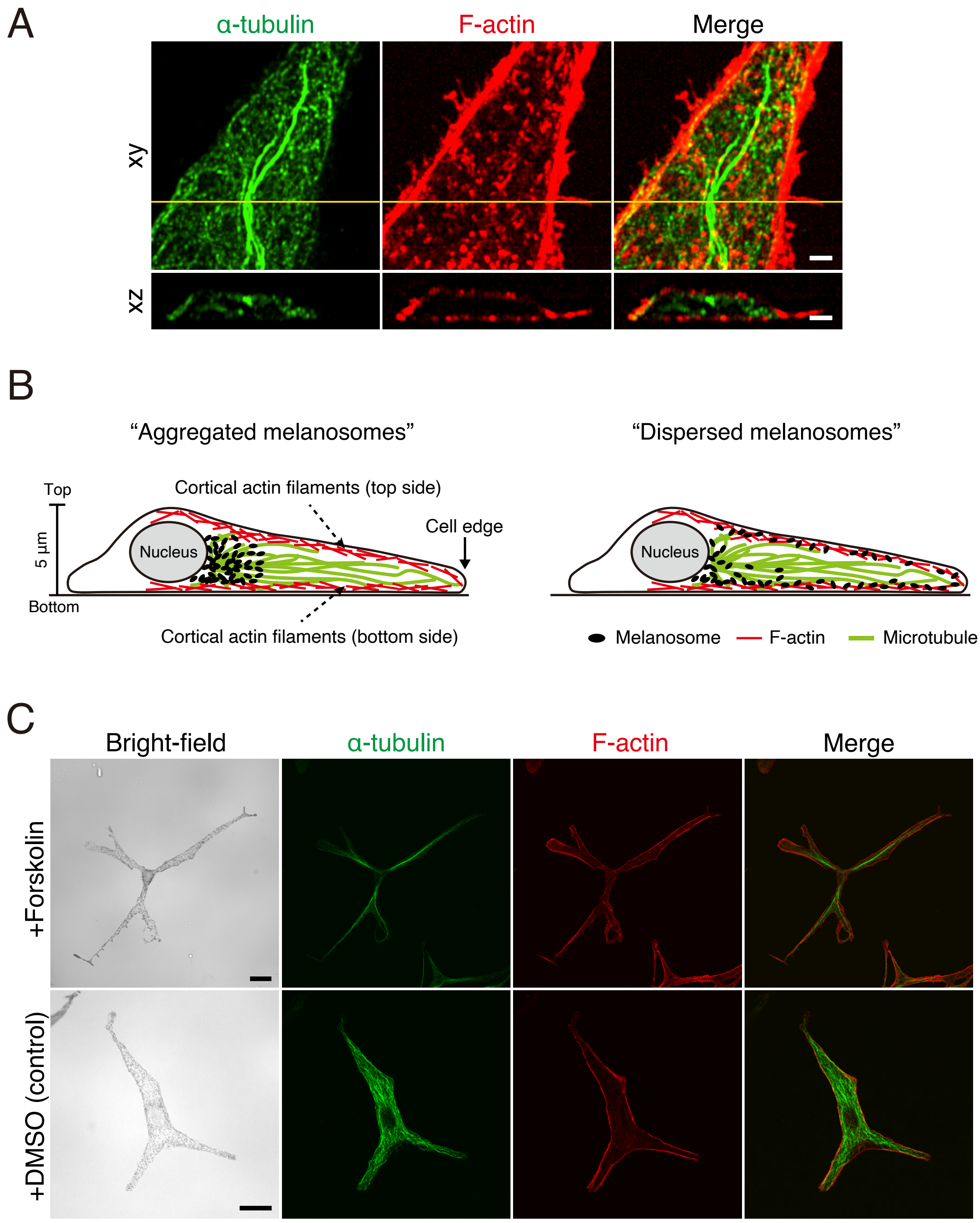

Fig. S2. Different spatial distributions of microtubules and actin filaments in melan-a cells. (A) Typical z-stack images of cytoskeletal distributions in melan-a cells. Horizonal sections of the z-stack images (yellow lines in the upper panels) are shown in the bottom panels. The cells were stained for α-tubulin (green) and F-actin (phalloidin, red). Scale bars, 2 µm. (B) Schematic models of 3D melanosome and cytoskeletal distributions in melanocytes. (C) Typical images of cytoskeletal distributions in dendritic melan-a cells. Melan-a cells were exposed to 20 µM forskolin or DMSO alone (control) for 20 h and stained for α-tubulin (green) and F-actin (phalloidin, red). Scale bars, 20 µm.

Journal of Cell Science • Supplementary information

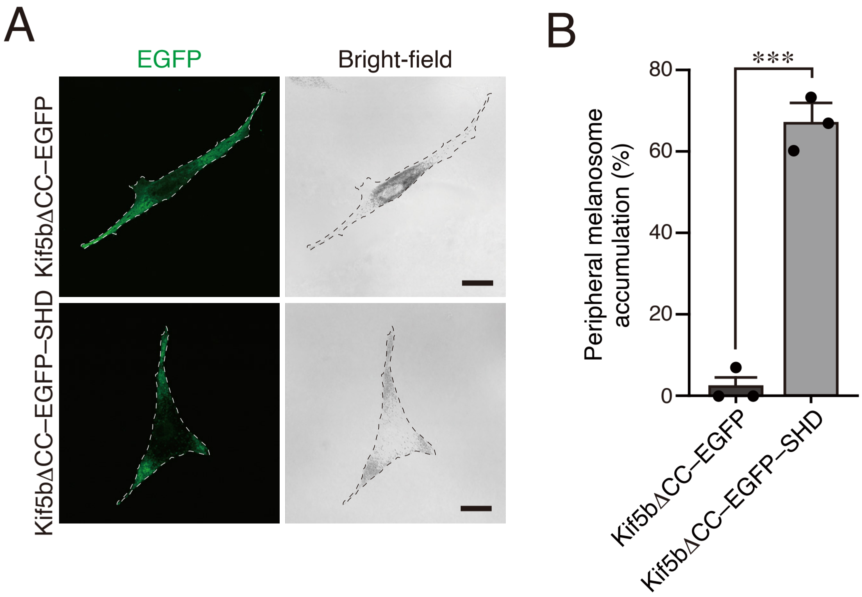

Fig. S3. Expression of Kif5bΔCC–EGFP–SHD, but not Kif5bΔCC–EGFP, in melan-ln cells induced peripheral melanosome accumulation. (A) Typical images of melan-ln cells (outlined with broken lines) expressing Kif5bΔCC–EGFP or Kif5bΔCC–EGFP–SHD. Note that melanosomes have accumulated at the edge of the melan-ln cell expressing Kif5bΔCC–EGFP–SHD, whereas the control Kif5bΔCC–EGFP cell still exhibited a perinuclear melanosome aggregation phenotype. Scale bars, 20 µm. (B) The percentages of cells exhibiting peripheral melanosome accumulation in (A). The error bars represent the means ± s.e.m. of data obtained in three independent experiments (n = 30 cells in each experiment). ***, P<0.001 (Student's unpaired t-test).

Journal of Cell Science • Supplementary information

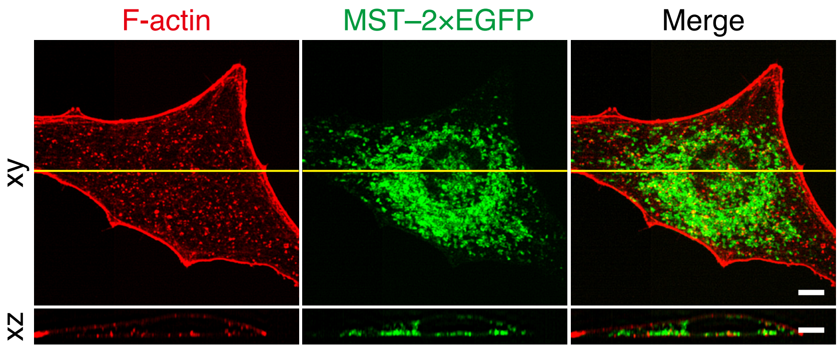

Fig. S4. Most of the aggregated melanosomes were present on the lower side of the melanocytes. Typical z-stack images of melan-a cells expressing TIR1(F74G)–P2A–AID–2×RBD27 and MST–2×EGFP (green). Horizonal sections of the z-stack images (yellow lines in the upper panels) are shown in the lower panels. The cells were stained for F-actin (phalloidin, red). Scale bars, 5 µm.

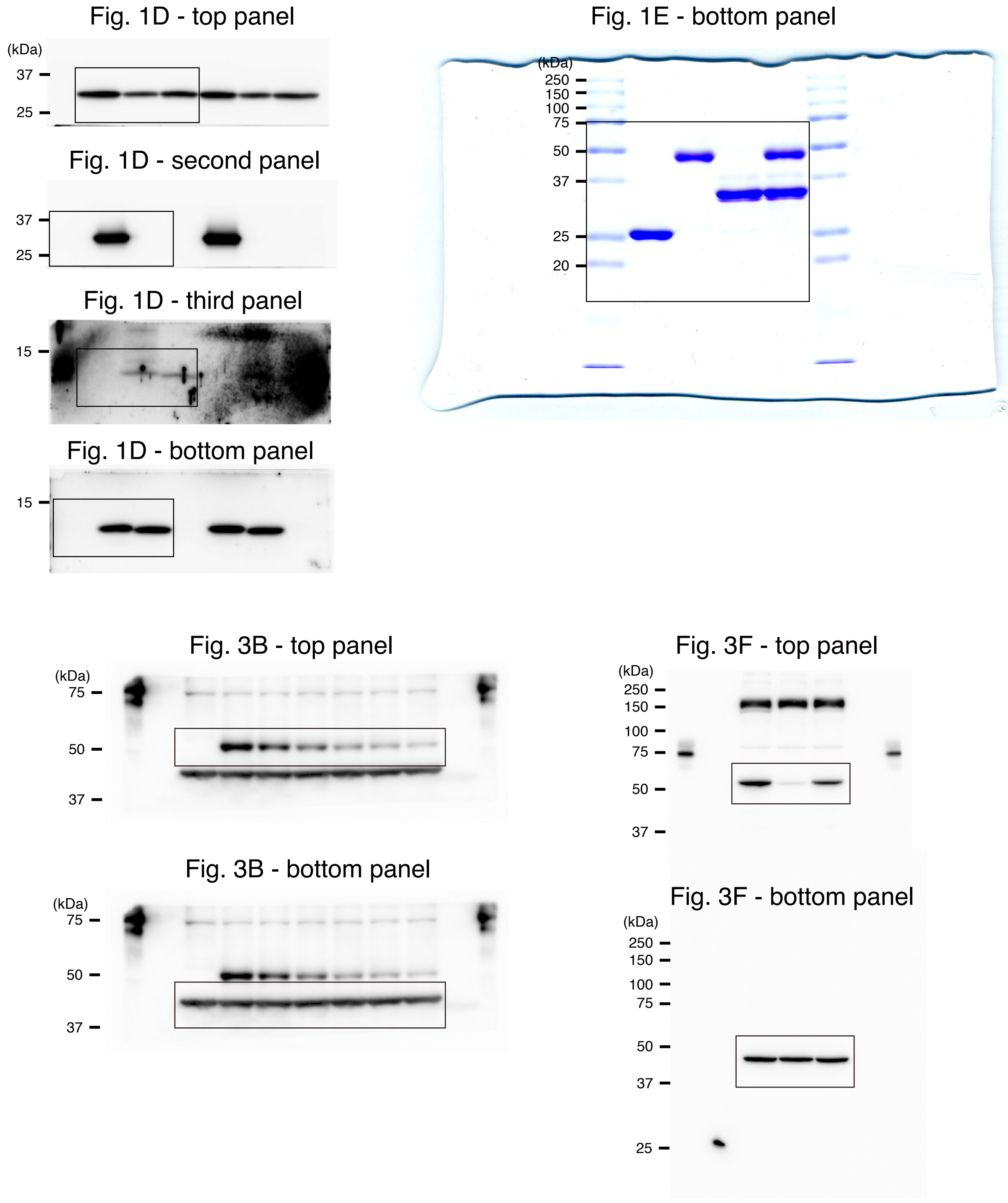

Fig. S5. Original data on the immunoblots and gels used in this study.

Journal of Cell Science • Supplementary information

Table S1. A list of materials used in study

**Oligonucleotides**	**Sequence** (5’-to-3’ direction)	**Source**
Primers for mutagenesis and construction		
mouse RBD27-5'	CTAAGATCTATGATCGACTTAAGTTTC	Nippon Gene (Toyama, Japan)
mouse RBD27-3’	GCGAATTCACTGCTCAGCTGCCGCTGG	Nippon Gene (Toyama, Japan)
rat RBD27-5’	CTAAGATCTATGATTGACTTAAGCTTC	Nippon Gene (Toyama, Japan)
rat RBD27-3’	CTACTCGAGCTGCTCAGCTGCTATCTG	Nippon Gene (Toyama, Japan)
rat RBD27-mut-5’	CTAAGATCTATGATTGACTTAAGCTTC<br>CTGACTGAAGAGGCACAAGAGGCCAT<br>CATGAAGGTTTTGCAGCGGGATGCTG<br>CTCTGAAGAGGGCCGAAGAAGAGGCA	Nippon Gene (Toyama, Japan)
TIR-BglII-Koz-Met	CTAAGATCTCCACCATGACGTACTTCC	Nippon Gene (Toyama, Japan)
TIR-L1-ΔStop-BamHI-NotI	CTAGCGGCCGCTAGGATCCTAGGATTT	Nippon Gene (Toyama, Japan)
TIR-L1-F74G-S	GGGAAGCCCCACGGCGCCGACTTCAA	Nippon Gene (Toyama, Japan)
TIR-L1-F74G-AS	GAGGTTGAAGTCGGCGCCGTGGGGCT	Nippon Gene (Toyama, Japan)

**Plasmids**	**RIKEN BioResource Research Center**	**Source**
pEF-FLAG-Rab27A	Cat#: RDB03423	*J. Biol. Chem.* (2002) **277**, 9212-9218
pGEX-4T-3-Rab27A	Cat#: RDB16851	*Genes Cells* (2006) **11**, 1023-1037
pGBD-C1-Rabs(CA)ΔCys	Cat#: RDB18802– RDB18924	*Mol. Cell. Proteomics* (2008) **7**, 1031-1042;<br>*J. Biol. Chem.* (2019) **294**, 6912-6922
pEF-T7-RBD27-WT	Cat#: RDB15090 (pmRFP-C1-mouse Slp2-a)	This study
pEF-T7-RBD27-mut (E11A/R32A)		This study (see also *Nat. Cell Biol.* (2004) **6**, 1195-1203)
pAct2-RBD27-WT		This study
pAct2-RBD27-mut (E11A/R32A)		This study
pEF-T7-GST-RBD27		This study
pEGFP-C1		Clontech-Takara Bio (Shiga, Japan) #6084-1
pEGFP-C1-2×RBD27	Cat#: RDB20906	This study
pEGFP-C1-2×RBD27-mut (E11A/R32A)	Cat#: RDB20907	This study
pEF-TIR1(F74G)-P2A-AID-2×RBD27	Cat#: RDB20908	This study
pEF-MST-2×EGFP	Cat#: RDB20909	This study
pEF-Kif5b-ΔCC-EGFP	Cat#: RDB20910	This study
pEF-Kif5b-ΔCC-EGFP-SHD	Cat#: RDB20911	This study

**Antibodies/Staining reagents**	**Dilution**	**Source**	**RRID**
anti-FLAG tag (M2)-HRP mouse mAb	IB (1/1,000 dilution)	Sigma-Aldrich (St. Louis, MA) A8592	AB_439702
anti-T7 tag-HRP mouse mAb	IB (1/1,000 dilution)	Novagen-Merck (Darmstadt, Germany) 69048	AB_11212778
anti-β-actin-HRP mouse mAb	IB (1/5,000 dilution)	Proteintech (Rosemont, IL) HRP-60008	AB_2819183
anti-Slp2-a SHD rabbit pAb	IB (0.9 µg/mL)	*J. Cell Sci.* (2004) **117**, 1945-1953	
anti-β-actin mouse mAb	IB (1/20,000 dilution)	MBL (Nagoya, Japan) G043	AB_2631287
anti-OsTIR1 rabbit pAb	IF (1/200 dilution)	MBL (Nagoya, Japan) PD048	AB_2909494
anti-α-tubulin mouse mAb	IF (1/100 dilution)	Sigma-Aldrich (St. Louis, MA) T6199	AB_477583
Alexa Fluor 488 Phalloidin	IF (1/500 dilution)	Invitrogen-Thermo Fisher Scientific (Waltham, MA) A12379	
PlasMem Bright Red	IF (1/100 dilution)	Dojindo (Kumamoto, Japan) P505	
Alexa Fluor 568 Phalloidin	IF (1/1000 dilution)	Invitrogen-Thermo Fisher Scientific (Waltham, MA) A12380	
anti-GFP rabbit pAb	IF (1/2000 dilution)	MBL (Nagoya, Japan) 598	AB_591816
Goat anti-Mouse IgG-Alexa Fluor 488	IF (1/500 dilution)	Invitrogen-Thermo Fisher Scientific (Waltham, MA) A32723	AB_2633275
Goat anti-Rabbit IgG-Alexa Fluor 488	IF (1/5000 dilution)	Invitrogen-Thermo Fisher Scientific (Waltham, MA) A32731	AB_2633280
Goat anti-Rabbit IgG-Alexa Fluor 555	IF (1/500 dilution)	Invitrogen-Thermo Fisher Scientific (Waltham, MA) A32732	AB_2633281
Goat anti-Rabbit IgG-Alexa Fluor 633	IF (1/500 dilution)	Invitrogen-Thermo Fisher Scientific (Waltham, MA) A21071	AB_141419

IB, immunoblot; IF, immunofluorescence; mAb, monoclonal antibody; pAb, polyclonal antibody.

Journal of Cell Science • Supplementary information

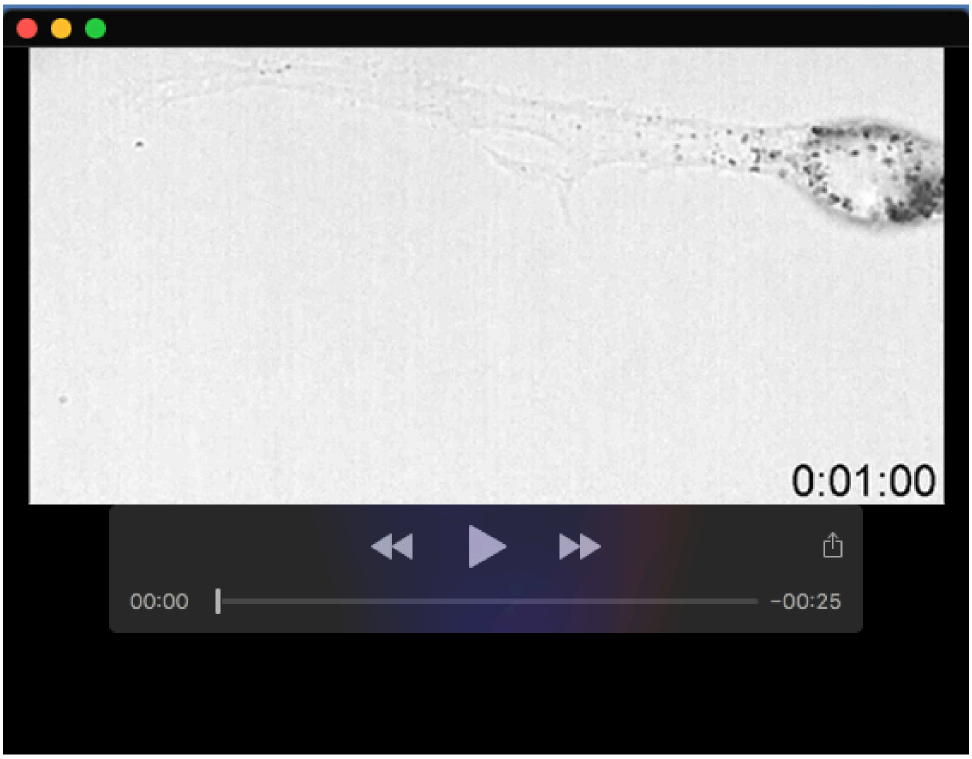

Movie 1. Melanosome movements in the dendrite of melan-a cells 0–20 min after the addition of 5-Ph-IAA.

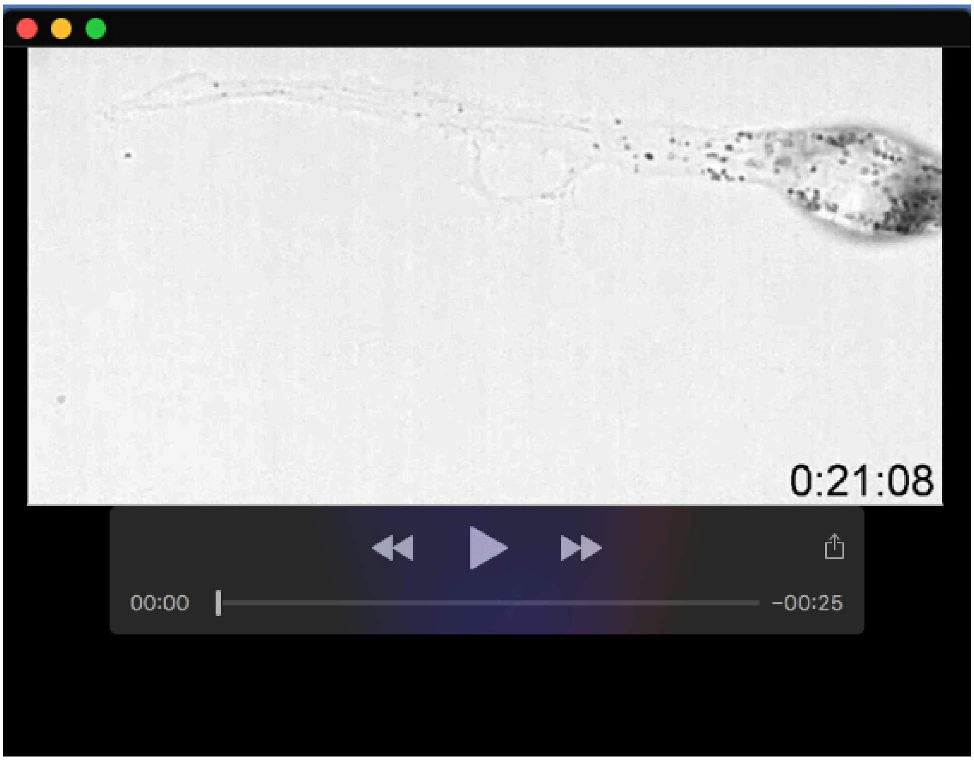

Movie 2. Melanosome movements in the dendrite of melan-a cells 20–120 min after the addition of 5- Ph-IAA.

Journal of Cell Science • Supplementary information
