# Supplementary material for: AID–2×RBD27, an auxin-inducible degron-based Rab27 trapper that reversibly inhibits the function of Rab27A in melanocytes
Source: J Cell Sci. 2025 Jun 10;138(11):jcs263878. doi: 10.1242/jcs.263878 (PMC12188314; doi:10.1242/jcs.263878)
Supplement: Supplementary file 1 [file joces-138-263878_review_history.pdf]

Journal of Cell Science

AID-2×RBD27, an auxin-inducible degron-based Rab27 trapper that reversibly inhibits the function of Rab27A in melanocytes

Akira Sugawara, Yuto Maruta and Mitsunori Fukuda
DOI: 10.1242/jcs.263878

Editor: Kathleen Green

Review timeline

Original submission:	25 January 2025
Editorial decision:	27 February 2025
First revision received:	9 April 2025
Accepted:	4 May 2025

Original submission

First decision letter

MS ID#: jcs.263878

MS TITLE: AID-2×RBD27, an auxin-inducible degron-based Rab27 trapper that reversibly inhibits the function of Rab27A in melanocytes

AUTHORS: Akira Sugawara; Yuto Maruta; Mitsunori Fukuda

ARTICLE TYPE: Tools and Resources

Dear Dr Fukuda,

We have now reached a decision on the above manuscript.

To see the reviewers' reports and a copy of this decision letter, please go to:

As you will see, the reviewers raise a number of substantial criticisms that prevent me from accepting the paper at this stage. They suggest, however, that a revised version might prove acceptable, if you can address their concerns. If you think that you can deal satisfactorily with the criticisms on revision, I would be pleased to see a revised manuscript. We would then return it to the reviewers.

Reviewer 1

Advance summary and potential significance to field

In this study, the authors demonstrated the auxin-inducible degron system is effective in cultured melanocytes by using cell biological and biochemical analyses, focused on the reversible regulation of Rab27A to evaluate the subcellular localization of melanosomes. Using the activated Rab27A-binding domain of Slp2-a (RBD27), the authors trapped endogenous Rab27A in melanocytes, preventing its interaction with effectors and thereby inhibiting its function, i.e. melanosome transport along AF. They then applied the AID-tagged RBD27 degradation system to reversibly restore melanosome transport, rescuing the defects caused by Rab27A inhibition, then removing the auxin analog restored Rab27A function, confirming the reversibility of the system in melanocytes.

© 2025. Published by The Company of Biologists under the terms of the Creative Commons Attribution License (https://creativecommons.org/licenses/by/4.0/).

Besides, 2D and 3D imaging analyses revealed melanosome localization and movement spatiotemporally. Using MST, a melanosomal marker, the authors demonstrated the precise spatial localization of melanosome moving along MT and AF. At the initial stage, melanosomes move rapidly in the anterograde direction by utilizing inner MTs and subsequently being trapped by cortical AFs. Furthermore, they showed melanosomes recruited into melanocytic dendrites move in a manner similar to that observed during transport from the perinuclear region toward periphery in the cell body. These are the primary findings by the authors and informative.
This study offers a clear demonstration of the spatiotemporal dynamics of melanosome movement, making it compelling and provides important insights into the still debated physiological functions of melanosomes' anterograde transport along MT and has potential for publication in the Journal of Cell Science.

Comments for the author

The manuscript is well written, easy to follow, and the data are clearly presented with a very few minor exceptions:

1) In Fig 3E, the auxin analog treatment only partially rescued the Rab27A inhibition phenotype (~50%). The authors should discuss the possible reason(s) for this incomplete rescue penetrance.

2) On page 9 line 50-51, the authors stated "melanosome distribution (10-30 min)..."
Fig 4 lacks data for the 10-min time point, so the basis for stating that is unclear to this reviewer.

3) In Fig 5E (right), the authors present quantitative data on the relationship between melanosome moving speed and intracellular position. The definition of "Slow" and "Fast" should be clearly stated in the legend.

<u>Reviewer 2</u>

Advance summary and potential significance to field

In this report, Sugawara et al. have developed a very effective tool to elegantly analyze melanosome movements and elucidate the role of Rab27 in various aspects of the trafficking of this organelle. This elegant study uses multiple approaches to analyze melanosome movements and sheds novel important insight on the mechanisms that regulate trafficking in melanocytes. Further, both the experimental approaches and tools developed can be broadly applicable to organelle trafficking in other systems. There are some areas that require attention, specifically related to lack of some important controls in the experiments. Specific concerns are listed below.

1. Fig. 1E: Although the experiments in Figures 1A-D convincingly show interactions between Rab27 and RBD, the in vitro pull-down experiments do not exclude the possibility that proteins in the COS-7 lysate associated with T7-GST-RBD may form a bridge between GST-RBD and GST-Rab27. Thus, this experiment does not unequivocally demonstrate a direct interaction between the two moieties.
2. In Fig. 2B, there appears to be RBD immunoreactivity in several cells around those outlined by the authors with dashed lines, although showing lower intensity than those outlined. Those cells also exhibit a punctate patter of GFP fluorescence, but do not show melanosome concentration around the nucleus. It appears that the level of RBD expression may modulate the phenotype, which needs to be addressed.
3. Unlike the statement on p. 7, L31, in Fig. 3B, there is still clearly detectable TIR1(F74G)-P2A-AID-2xRBD27 two h after treatment.
4. Fig. 3C shows changes in melanosome distribution, but the authors need to demonstrate that this cell also expresses TIR1(F74G)-P2A-AID-2xRBD27. Similar considerations apply to Figs. 4, 5. The criteria used to identify cells expressing the exogenous proteins for further analysis need to be included.
5. The organization of the microtubules in the single cell expressing TIR1(F74G)-P2A-AID-2xRBD27 (Fig. S1) appears to be substantially different from those shown in Control or CytD-treated

© 2025. Published by The Company of Biologists under the terms of the Creative Commons Attribution License (https://creativecommons.org/licenses/by/4.0/).

cells. This needs to be addressed. Further, images of several transfected melanocytes demonstrating their microtubule organization need to be included.

6. The plasma membrane marker shown in Figures 5D and 5E needs to be identified, as it is not clear whether this stain specifically labels the cell membrane. Given that these data also include cells expressing TIR1(F74G)-P2A-AID-2xRBD27, the analysis of Fig. 5D and E should include melanosome movements in untransfected cells, for comparison.

7. The experiment of Fig. S2C needs to include images of more than one single cell, and, additionally, appropriate vehicle control cells.

Minor comments:

1. On p. 4, L. 9-10, the authors state that "…no … tools that reversibly inhibit Rab functions have ever been developed". Although they may not have been reported to-date, this does not necessarily indicate that they have never developed.
2. On p. 5, L39, the E11A/R32A mutant did not impair Rab27 binding ability. Rather, this mutant was incapable of binding to Rab27.
3. P. 7, L. 31 reads "…120 min later treatment", but should read "... 120 min after treatment".
4. The X axis label of Fig. S2B should read "Bottom", not "Btottom".

First revision

Author response to reviewers' comments

[Response to the reviewer #1]

In this study, the authors demonstrated the auxin-inducible degron system is effective in cultured melanocytes by using cell biological and biochemical analyses, focused on the reversible regulation of Rab27A to evaluate the subcellular localization of melanosomes. (. . . . . . .) This study offers a clear demonstration of the spatiotemporal dynamics of melanosome movement, making it compelling and provides important insights into the still debated physiological functions of melanosomes' anterograde transport along MT and has potential for publication in the Journal of Cell Science.

First of all, we thank the reviewer for spending time and effort in reviewing our manuscript and for his/her very positive comments.

Suggestions to authors:

1. In Fig 3E, the auxin analog treatment only partially rescued the Rab27A inhibition phenotype (~50%). The authors should discuss the possible reason(s) for this incomplete rescue penetrance.

As suggested, we have described the possible reason for the incomplete rescue effect of 5-Ph-IAA on peripheral melanosome distribution in the revised manuscript (pages 8): the incomplete rescue effect of 5-Ph-IAA on peripheral melanosome distribution is presumably caused by the presence of undegraded AID– 2×RBD27 (see Fig. 3B, lane 7), which still inhibits the function of Rab27A in melanosome transport.

2. On page 9 line 50-51, the authors stated "melanosome distribution (10-30 min). " Fig 4 lacks data for the 10-min time point, so the basis for stating that is unclear to this reviewer.

We apologize for this mistake. “0 and 30 min” are correct.

3. In Fig 5E (right), the authors present quantitative data on the relationship between melanosome moving speed and intracellular position. The definition of "Slow" and "Fast" should be clearly stated in the legend.

As pointed out by the reviewer, we have included the definition of slow and fast

© 2025. Published by The Company of Biologists under the terms of the Creative Commons Attribution License (https://creativecommons.org/licenses/by/4.0/).

speed of melanosome transport in the revised figure legend. In brief, we have defined more than 0.3 μm/sec of melanosome transport as the fast speed.

[Response to the reviewer #2]

In this report, Sugawara et al. have developed a very effective tool to elegantly analyze melanosome movements and elucidate the role of Rab27 in various aspects of the trafficking of this organelle. This elegant study uses multiple approaches to analyze melanosome movements and sheds novel important insight on the mechanisms that regulate trafficking in melanocytes. Further, both the experimental approaches and tools developed can be broadly applicable to organelle trafficking in other systems. There are some areas that require attention, specifically related to lack of some important controls in the experiments. Specific concerns are listed below.

We thank the reviewer for spending time and effort in reviewing our manuscript and for his/her very helpful suggestions. According to the reviewer's suggestions, we have revised the manuscript as described below.

1. Fig. 1E: Although the experiments in Figures 1A-D convincingly show interactions between Rab27 and RBD, the in vitro pull-down experiments do not exclude the possibility that proteins in the COS-7 lysate associated with T7-GST-RBD may form a bridge between GST-RBD and GST-Rab27. Thus, this experiment does not unequivocally demonstrate a direct interaction between the two moieties.

We agreed with the reviewer. In our direct binding assays, however, we tried to remove unrelated proteins (or RBD27-binding proteins) in COS-7 cell lysates from anti-T7 tag antibody-conjugated agarose beads by washing the beads three times with a 250 mM NaCl-containing buffer (50 mM HEPES-KOH, pH 7.2, 1 mM $MgCl_2$, and 0.1% Triton X-100) beforehand. The purity of T7–GST–RBD27 was verified both by CBB staining and by immunoblotting with anti-T7 tag antibody (see Reviewers only Fig. A). Although several faint minor bands in addition to the major T7-GST-RBD27 band were observed by CBB staining, all these bands were detected with anti-T7 tag antibody (arrows in lanes 2 and 4). We thus thought that these minor bands are likely to be degradation products or post-translationally modified form of T7-GST-RBD27.

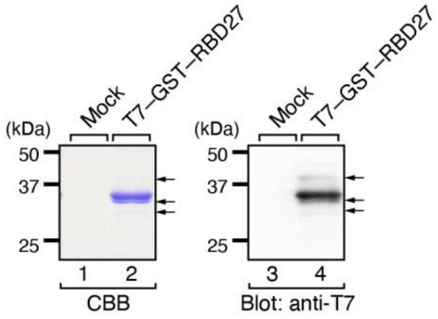

Reviewers only Fig. A. Purity of purified T7-GST-RBD27 from COS-7 cell lysates.

Since the previous Fig. 1E data contained relatively large amount of degradation products, we have replaced them with better data in the revised manuscript. In addition, we have described the purification protocol of T7-GST-RBD27 in more detail in the revised method section (pages 17–18).

2. In Fig. 2B, there appears to be RBD immunoreactivity in several cells around those outlined by the authors with dashed lines, although showing lower intensity than those outlined. Those cells also exhibit a punctate patter of GFP fluorescence, but do not show melanosome concentration around the nucleus. It appears that the level of RBD expression may modulate the phenotype, which needs to be addressed.

As pointed out by the reviewer, we previously showed that melan-a cells with high

© 2025. Published by The Company of Biologists under the terms of the Creative Commons Attribution License (https://creativecommons.org/licenses/by/4.0/).

EGFP–Slp2-a-SHD (i.e., RBD27) expression levels exhibited aggregated melanosomes, whereas cells with low expression levels exhibited the normal peripheral melanosome distribution (see Fig. 2a in Kuroda and Fukuda (2004) *Nat. Cell Biol.* 6, 1195–1203). Since EGFP–2×RBD27 acts as a dominant negative construct, it is necessary to trap all the Rab27A molecules on melanosomes to induce a perinuclear aggregation phenotype. To avoid confusion, we have also indicated low EGFP– 2×RBD27-expressing cells with the normal peripheral melanosome distribution (as blue asterisks in Fig. 2B) and described a possible reason in the revised figure legend.

3. Unlike the statement on p. 7, L31, in Fig. 3B, there is still clearly detectable TIR1(F74G)-P2A-AID-2xRBD27 two h after treatment.

We have changed the sentence as follows: more than 80% of the AID– 2×RBD27 had disappeared 120 min after treatment, although the AID-2×RBD27 band was still visible (Fig. 3B).

4. Fig. 3C shows changes in melanosome distribution, but the authors need to demonstrate that this cell also expresses TIR1(F74G)-P2A-AID-2xRBD27. Similar considerations apply to Figs. 4, 5. The criteria used to identify cells expressing the exogenous proteins for further analysis need to be included.

We also think that this is an important issue. Actually, in some cases, we have immunostained the cells that had been used for live-cell imaging with anti-TIR1 antibody to verify whether TIR1(F74G)-P2A-AID-2×RBD27 was actually expressed in them. As expected, cells that showed peripheral melanosome dispersion in response to 5-Ph-IAA contained TIR1 (see Reviewers only Fig. B). We thus believe that cells showing 5-Ph-IAA-dependent peripheral melanosome dispersion in Figs. 3C, 4, and 5 should express TIR1(F74G)-P2A-AID-2×RBD27. We have included this information in the revised method section (page 20).

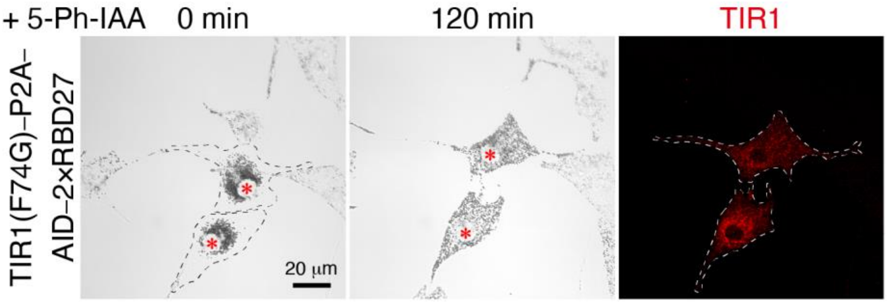

Reviewers only Fig. B. Expression of TIR1 in cells showing 5-Ph-IAA-dependent peripheral melanosome dispersion after live-cell imaging.

5. The organization of the microtubules in the single cell expressing TIR1(F74G)-P2A-AID-2xRBD27 (Fig. S1) appears to be substantially different from those shown in Control or CytD-treated cells. This needs to be addressed. Further, images of several transfected melanocytes demonstrating their microtubule organization need to be included.

As suggested, we have replaced cytoskeletal images in TIR1(F74G)–P2A– AID–2xRBD27-expressing cells with better images in the revised manuscript. Three additional typical images were also shown below (Reviewers only Fig. C).

© 2025. Published by The Company of Biologists under the terms of the Creative Commons Attribution License (https://creativecommons.org/licenses/by/4.0/).

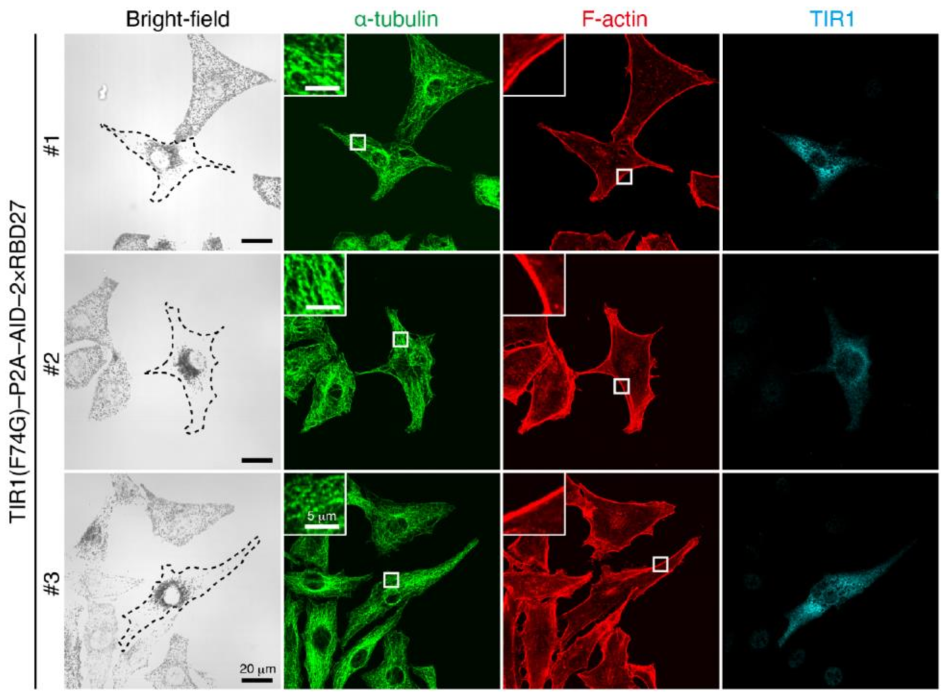

Reviewers only Fig. C. Distribution of microtubules in TIR1(F74G)–P2A–AID– 2xRBD27-expressing cells.

6. The plasma membrane marker shown in Figures 5D and 5E needs to be identified, as it is not clear whether this stain specifically labels the cell membrane. Given that these data also include cells expressing TIR1(F74G)-P2A-AID-2xRBD27, the analysis of Fig. 5D and E should include melanosome movements in untransfected cells, for comparison.

We apologize that we did not describe the plasma membrane marker in the original manuscript. We have used a commercially available plasma membrane staining dye, PlasMem Bright Red (P505; DOJINDO Laboratories, Kumamoto, Japan) in the present study. We have included this information in the revised manuscript (page 20).

We think that melanosome movements in untransfected cells are not so meaningful, because most of the melanosomes had already been transported to the peripheral area of cells (and may be tethered to the actin filaments), and anterograde melanosome transport on microtubules was rarely observed. So, that's why we have developed the AID–2×RBD27 system to analyze anterograde melanosome transport.

7. The experiment of Fig. S2C needs to include images of more than one single cell, and, additionally, appropriate vehicle control cells.

In Fig. S2C, we wanted to show the distinct distributions of microtubules and actin filaments in forskolin-treated dendritic melanocytes. When several dendritic cells are shown at low magnification, it is difficult to observe distinct cytoskeletal distributions (see Reviewers only Fig. D). Thus, we would like to show a single dendritic cells as it is. As suggested, however, we have included a vehicle control in the revised manuscript.

© 2025. Published by The Company of Biologists under the terms of the Creative Commons Attribution License (https://creativecommons.org/licenses/by/4.0/).

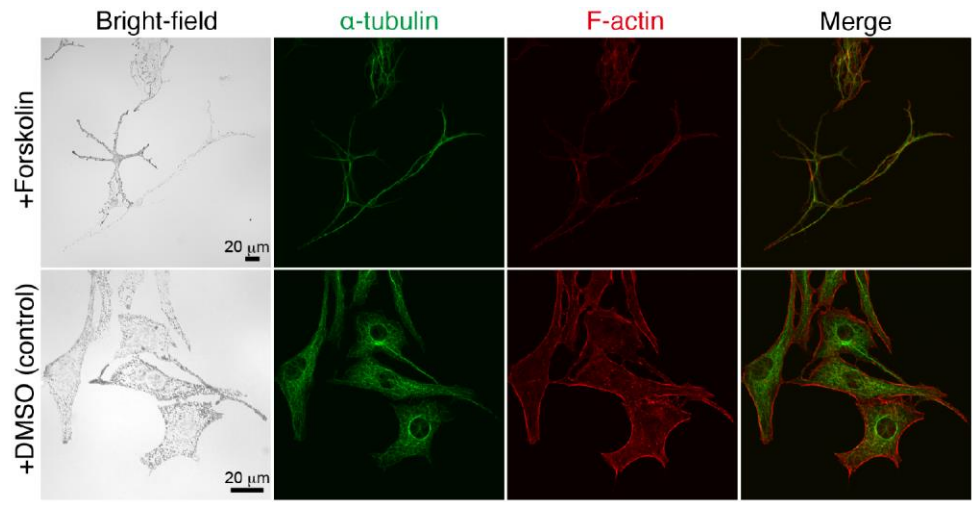

Reviewers only Fig. D. Low magnification images of forskolin (Fsk)-treated dendritic melanocytes.

Minor comments:

1. On p. 4, L. 9-10, the authors state that "...no ... tools that reversibly inhibit Rab functions have ever been developed". Although they may not have been reported to-date, this does not necessarily indicate that they have never developed.

As suggested, we have modified the sentence as follows: no easy-to-use tools that "reversibly" inhibit Rab functions are currently available.

2. On p. 5, L39, the E11A/R32A mutant did not impair Rab27 binding ability. Rather, this mutant was incapable of binding to Rab27.

As suggested, we have corrected the sentence in the revised manuscript.

3. P. 7, L. 31 reads "...120 min later treatment", but should read "... 120 min after treatment".

Thank you for pointing it out. We have corrected this typo in the revised manuscript.

4. The X axis label of Fig. S2B should read "Bottom", not "Btottom".

Thank you for pointing it out. We have corrected the labeling in Fig. S2B.

Second decision letter

MS ID#: jcs.263878R1

MS Title: AID-2×RBD27, an auxin-inducible degron-based Rab27 trapper that reversibly inhibits the function of Rab27A in melanocytes

Authors: Akira Sugawara; Yuto Maruta; Mitsunori Fukuda

Article Type: Tools and Resources

Dear Dr Fukuda,

I am happy to tell you that your manuscript has been accepted for publication in Journal of Cell Science, pending standard publication integrity checks.

Reviewer 1

© 2025. Published by The Company of Biologists under the terms of the Creative Commons Attribution License (https://creativecommons.org/licenses/by/4.0/).

Advance summary and potential significance to field

Comments for the author

The authors have addressed the minor concerns raised by the reviewer. No major issues remain.

© 2025. Published by The Company of Biologists under the terms of the Creative Commons Attribution License (https://creativecommons.org/licenses/by/4.0/).
